# Evaluating the effectiveness of the HPV vaccination programme in England, using regression discontinuity design

**DOI:** 10.23889/ijpds.v9i5.2689

**Published:** 2024-09-18

**Authors:** Isobel Ward, Charlotte Bermingham, Kate Soldan, Vahe Nafilyan

**Affiliations:** 1 Office for National Statistics; 2 UK Health Security Agency

## Background

In England, the National human papillomavirus (HPV) HPV Immunisation Programme was introduced for young girls in 2008. Whilst clinical trials have shown high vaccine effectiveness, real-world evidence is still accruing. Estimating vaccine effectiveness based on real-world data without randomised control trials is challenging, but critical for policy makers. Using Census 2011 data and a population level linked dataset, we exploit a regression discontinuity design (RDD) to assess the impact of the catch-up vaccination programme on cervical dysplasia and cancer diagnoses as recorded in hospital and mortality data in girls born between 1987 and 1992.

## Results

We estimate that vaccination reduces the incidence of cervical dysplasia diagnoses by 27% and cervical cancer diagnoses by 75% respectively. Girls were followed for a 7-year period between ages 23 and 30 years in girls who were offered vaccination at ages 17 to 18 years as part of the catch-up cohort compared to those who were not eligible for HPV vaccination.

## Conclusions and Implications

Our work demonstrates how pivotal the HPV vaccination programme has been for reducing adverse cervical outcomes in young women. Additionally, it shows that the estimates of the effectiveness of HPV vaccination obtained using a RDD are comparable to those based on more classic observational studies. This work shows real world vaccination effectiveness and demonstrates how pivotal the national HPV campaign has been in young girls.

